# Socio-economic characterisation of date palm (*Phoenix dactylifera* L.) growers and date value chains in Pakistan

**DOI:** 10.1186/s40064-016-2855-4

**Published:** 2016-08-02

**Authors:** Ghayoor Fatima, Iqrar Ahmad Khan, Andreas Buerkert

**Affiliations:** 1 Organic Plant Production and Agroecosystems Research in the Tropics and Subtropics, University of Kassel, Witzenhausen, Germany; 2 Institute of Horticultural Sciences, University of Agriculture, Faisalabad, 38040 Pakistan

**Keywords:** Date palm cultivars, Food security, Income strategies, Stakeholders

## Abstract

Increasing food production to feed its rapidly growing population is a major policy goal of Pakistan. The production of traditional staples such as rice (*Oryza sativa* L.) and bread wheat (*Triticum aestivum* L.) has been intensified in many regions, but not in remote, drought-ridden areas. In these arid, marginal environments dates and their by-products are an option to complement staples given their high nutritive value and storability. To fill knowledge gaps about the role of date palm in the household (HH) income of rural communities and the structure of date value chains, this project studied date palm production across six districts in four provinces of Pakistan. During 2012–2013 a total of 170 HHs were interviewed with a structured questionnaire using a snowball sampling approach. The results showed that most of the HH were headed by males (99 %) who were married (74 %) and often illiterate (40 %). Agriculture was the main occupation of date palm growers (56 %), while a few coupled agricultural activities with business (17 %) or extra-farm employment opportunities (government 9 %; private sector 8 %). Date sales contributed >50 % to the total income of 39 % of HH and 90–100 % to 24 % of HH. Overall farmers grew a total of 39 date palm cultivars and cultivated an average of 409 ± 559 mature date palms. The majority of the respondents sold dates to commission agents (35 %), contractors (22 %) and wholesalers (21 %), while 28 % of HH cultivated date palms only for self-consumption. Date palm growers had only limited knowledge about high quality date cultivars, optimized farm management and about effective post-harvest conservation. Changes in extension and marketing efforts are needed to allow farmers to better exploit value chains in date thereby reaping higher benefits from improved market access to secure their often marginal income.

## Background

In 2012, global date production amounted to about 7 million t with a total market value exceeding 1 billion US$ whereby the top six date producing countries accounting for 75 % of total production. Pakistan currently is the sixth largest date producer with an annual production of 600,000 t in 2012 and an increase in its cultivation area from 41,240 ha in 1992 to 95,000 ha in 2012 (FAO 2014). More than 300 date varieties are known to exist in the country of which the twelve most commercially important cultivars are: Karbalaen, Aseel, Muzawati, Fasli, Begum Jhangi, Halawi, Dashtiari, Sabzo, Koharba, Jaan Swore, Rabai, and Dhakki (Ata 2011).

In Pakistan date palm has a primary importance as a subsistence crop, particularly in its vast desert areas (Hassan et al. 2006; Ata et al. 2012). However, dates are also marketed all over the country as a highly appreciated fruit and confectionery product of which use peaks during the Muslim feast of Ramadan and the Hindu celebration of Diwali. The mineral, carbohydrate, and vitamin rich dates are an excellent source of food not only for humans, but low grade parts of the harvest also serve as a feed supplement for livestock at times of scarcity (Zohary and Hopf 2000; Al-Shahib and Marshall 2003; Hassan et al. 2006).

Date palm is dioecious in nature with separate male and female trees that are usually wind pollinated, but insect pollination also occurs. However, economic fruit production is only possible if 60–80 % of the female flowers are manually pollinated (Nixon and Carpenter 1978; Zaid and de Wet 2002) whereby pollen affects the shape and size of date palm seeds. Pollen also has a metaxenia effect affecting the shape and size of the fruit, time of ripening and fruit development (Nixon 1934, 1936). Many date varieties produce fruits even without pollination, but those are slender in size with imperfect seed (without embryo and endosperm) and ripen very late (Swingle 1928).

In Pakistan date palm is cultivated in a wide range of cropping and farming systems such as oases, groves, home gardens, as a mono-crop and as an intercrop (Abouziena et al. 2010; Bhansali 2010).

Date palm has numerous uses and yields many useful products for humans (Chao and Krueger 2007). Leaves are used for making roofs, mats, staple dishes, hand fans, baskets, packaging material, and also for ropes and fences (PHDEB 2008). Trunks can be used to construct houses and bridges, and as packing material for local transportation of vegetables and fruits (Anwar 2006). Terminal buds and young leaves can be cooked as vegetables while rachises are used for paper making (El Hadrami and Al Khayri 2012; Khiari et al. 2011). Moreover, date cultivation and production offers many jobs in groves during fruit harvest and processing (Jain 2012).

The socio-economic conditions and food security status in date palm growing areas in Pakistan are poorly documented (Suleri and Haq 2009). Some authors claim that rural income in rural Pakistan can be raised by the on-farm exploitation of more efficient post-harvest and marketing of dates (Goletti and Samman 1999). Date growers in Pakistan often depend on advanced payments and other informal credits from commission agents, wholesalers and contractors who skim off a substantial portion of the fruit´s final market value (Ata 2011). Date palm growers are often limited in self-marketing of their products given their poor education (Khushk et al. 2009). The prevalence of traditional marketing structures results in an estimated 30–40 % deterioration of fresh produce before it reaches the consumer (PHDEB 2008). Other limitations in date palm cultivation areas include low quality of date palm cultivars, poor farm management, lack of processing facilities, uncertainty in prices at the time of selling and shortage of qualified trained labour (Mahmoudi et al. 2008).

In view of the above this study was conducted to fill knowledge gaps about the value chain of date in Pakistan and its potential role in income strategies of poor people in the country´s arid marginal areas, both as a subsistence crop and a market commodity and to develop recommendations for strengthening dates´ contribution to daily income for rural HHs.

## Methods

### Study area and data collection

For this study six districts within four provinces of Pakistan with large areas under date palm cultivation were selected: Jhang, Muzaffargarh and Bahawalpur districts in the Punjab province, Dera Ismail Khan (D. I. Khan) district in Khyber Pakhtunkhwa (KPK), Khairpur in Sindh, and Panjgur in Baluchistan (Table 1). Apart from date palm, these districts produce a wide variety of annual field crops and perennials (trees and shrubs; Table 2).

**Table 1:** Biophysical characteristics of the six date producing districts in the tropical desert climate zone during 2012–2013. *Source*: adapted from Beinroth et al. (1985); www.namc.pmd.gov.pk/agromet-bulletins.php#

District name	Longitude (E)	Latitude (N)	Soil type	Average annual temperature (°C)	Average annual precipitation (mm)	Summer climate	Winter climate
Jhang	72°15′00″	31°25′00″	Loamy, clayey and sandy	24.7	180	Hot and dry	Cold and dry
Bahawalpur	67°43′00″	26°49′00″	Loamy, clayey and sandy	25.7	200	Very hot and dry with frequent dust storms	Cold and dry
Muzaffargarh	71°04′60″	30°19′60″	Loamy and clayey	25.6	127	Very hot with dust storms	Arid and mild
Dera Ismail Khan	72°19′41″	31°83′14″	Loam to clay loam	24.5	249	Hot desert	Mild
Khairpur	68°45′26″	27°31′50″	Loamy, clayey and sandy	26.9	178	Very hot and sunny	Mild to warm
Panjgur	64°06′00″	26°58′00″	Loamy and partly gravelly	21.7	109	Hot	Cold

*PMD* Pakistan Meteorological Department (accessed on 12 February 2015)

**Table 2:** Major annual and perennial field crops and fruits produced by 170 date palm growers in six districts of Pakistan, interviewed during 2012–2013. *Source*: adapted from Malik (1994)

District name	Annual field crops	Annual and perennial fruits
Jhang	Wheat (*Triticum aestivum* L.) Cotton (*Gossypium hirsutum* L.) Sugarcane (*Saccharum officinarum* L.) Rice (*Oryza sativa* L.) Maize (*Zea mays* L.)	Mango (*Mangifera indica* L.) Dates (*Phoenix dactylifera* L.) Ber (*Ziziphus jujuba* Mill.)
Bahawalpur	Wheat (*Triticum aestivum* L.) Cotton (*Gossypium hirsutum* L.) Rice (*Oryza sativa* L.) Sugarcane (*Saccharum officinarum* L.) Sunflower (*Helianthus annuus* L.) Mustard (*Brassica napus* L.)	Mango (*Mangifera indica* L.) Citrus (*Citrus* L.) Dates (*Phoenix dactylifera* L.) Guava (*Psidium guajava* L.)
Muzaffargarh	Wheat (*Triticum aestivum* L.) Cotton (*Gossypium hirsutum* L.) Sugarcane (*Saccharum officinarum* L.) Rice (*Oryza sativa* L.) Sunflower (*Helianthus annuus* L.) Mustard (*Brassica napus* L.)	Mango (*Mangifera indica* L.) Citrus (*Citrus* L.) Dates (*Phoenix dactylifera* L.) Pomegranate (*Punica granatum* L.)
Dera Ismail Khan	Wheat (*Triticum aestivum* L.) Sugarcane (*Saccharum officinarum* L.) Gram (*Vigna mungo* (L.) Hepper) Sorghum (*Sorghum bicolor* (L.) Moench) Millet (*Pennisetum americanum* (L.) R.Br.)	Watermelon (*Citrullus lanatus* (Thunb.) Matsum. & Nakai) Mango (*Mangifera indica* L.) Dates (*Phoenix dactylifera* L.)
Khairpur	Wheat (*Triticum aestivum* L.) Cotton (*Gossypium hirsutum* L.) Sugarcane (*Saccharum officinarum* L.) Rice (*Oryza sativa* L.)	Mango (*Mangifera indica* L.) Citrus (*Citrus* L.) Dates (*Phoenix dactylifera* L.) Banana (*Musa* × *paradisiaca* L.)
Panjgur	Wheat (*Triticum aestivum* L.) Rice (*Oryza sativa* L.) Barley (*Hordeum vulgare* L.)	Almonds (*Prunus dulcis* (Mill.) D.A.Webb) Pomegranate (*Punica granatum* L.) Grapes (*Vitis vinifera* L.) Peaches (*Prunus persica* Siebold & Zucc.) Pistachios (*Pistacia vera* L.) Dates (*Phoenix dactylifera* L.)

**Fig. 1:**
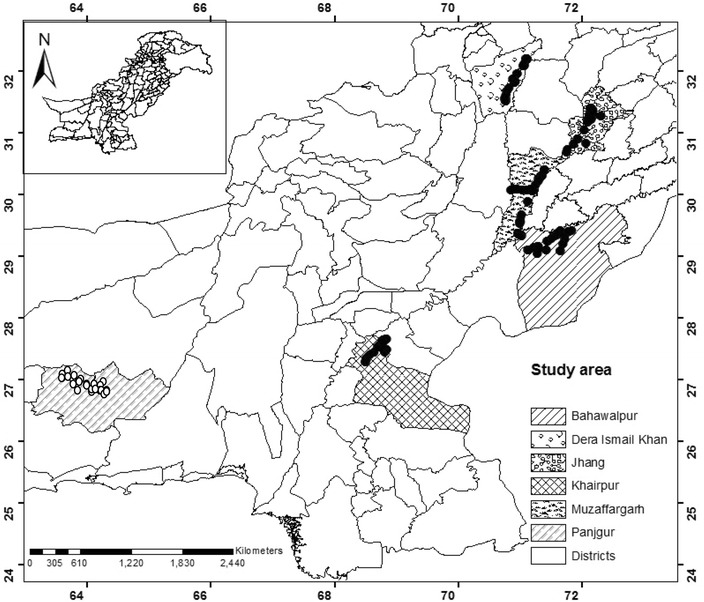
GIS-based map of the study area with the position of households in Pakistan, interviewed during 2012–2013. *Black dots* mark the household’s location in the three provinces; Punjab, Khyber Pakhtunkhwa, and Sindh. *White dots* represent the rough locations of interviewed households in Baluchistan province, since recording of GPS coordinates was not allowed in this region

From June 2012 to October 2013 a structured questionnaire with open-ended and closed questions was used for face-to-face interviews with 170 date palm growers (Fig. 1). The questionnaire had been pre-tested with 14 farmers and modified as required; it covered the following major areas: (1) demographic HH aspects (age, education, marital status), (2) list of income and expenditures, (3) date palm cultivation and distribution in the field, (4) structures of value chains, and (5) irrigation sources and crops grown in and around palm groves.

A snowball sampling scheme (Goodman 1961; Dossa et al. 2015) was employed whereby in each settlement the first interviewed HH growing date palm, selected by a local guide, was requested to provide names and addresses of three other HHs in his locality that cultivated date palm. From these three names, one was randomly selected for the next interview. The questions were orally translated from English into the local languages (Urdu, Punjabi, Saraiki, Balochi, and Sindhi) and translated replies noted down in English by the interviewer. One interview lasted 50–90 min. Interviews were also conducted with stakeholders involved in the supply chain of dates in the area. Stakeholders were selected with the help of growers, who were linked to the former by harvesting contracts. After each interview, the location of the HH was recorded with a handheld GPS device (GARMIN^®^ Vista HCxe Trex, GARMIN International, Inc., Olathe, KS, USA), except for Panjgur district in Baluchistan, where security concerns prevented the use of GPS devices and position estimates were based on Google Earth satellite images.

To estimate date palm contribution to the HH income, fresh fruit weight was measured and multiplied with the local price of dates per kg. To determine fresh fruit weight per date palm, the number of bunches was counted, from three of them dates were separated from the rachis, weighted, and subsequently average bunch weight was multiplied with the number of bunches per date palm.

### Statistical analysis

The data were coded into numerical values, tabulated and subjected to descriptive analysis in Excel (Microsoft Office 2007). Initially the five most important variables (province-wise distribution of date palm cultivars, number of mature date palms, planting types, yield, and selling of dates) that characterized date palm growers were subjected to Spearman correlation analysis and key variables least correlated with each other were identified (Raza et al. 2014). Subsequently, a two-step cluster analysis was performed comprising both categorical and numeric data on socio-economic household characteristics (Abas et al. 2013; Abdulkadir et al. 2012; Dossa et al. 2011). Kruskal–Wallis tests and Chi^2^ analyses were used to detect significant differences and to evaluate the effect of assessed variables (categorical and continuous) on the clusters (Vazhacharickal et al. 2013; Rehman et al. 2013). Because of the large differences in the financial status (land and labour assets, income and expenses) of HHs, they were divided into seven groups (Rehman et al. 2013). All statistical analyses were performed with SPSS version 17.0 (SPSS Inc., Chicago, IL, USA). The significance threshold was set at P ≤ 0.05.

## Results

### Grower characteristics

Most agricultural production in date palm growing was supervised by married, poorly educated, medium-aged male family heads with large families (Table 3).

**Table 3:** Household (HH) characteristics of 170 date palm growers in four provinces (Punjab, Khyber Pakhtunkhwa, Sindh, and Baluchistan) of Pakistan, interviewed during 2012–2013

Variable	Frequency (n)	Percentage
*Marital status*		
Single	11	6
Married	125	74
Widowed	29	17
Divorced	5	3
*Education*		
Illiterate	68	40
Primary	29	17
Secondary	40	24
Intermediate	10	6
Bachelor	14	8
Master	9	5
*Average age of HH head (years)*		
≤30	8	5
31–55	105	62
>55	57	33
*Type of date palm grower*		
Small scale	121	71
Medium scale	30	18
Large scale	19	11

Based on the two-step cluster analysis, three categories of date palm growers were identified: (1) small scale (SS) date palm growers cultivating an average of 103 (±99) mature date palms accounted for 71 % of all HHs; (2) medium scale (MS) growers with 812 (±237) mature date palms represented 18 % of the HHs, and (3) large scale (LS) growers with 1721 (±244) mature date palms made up 11 % of the HHs (Table 3).

**Fig. 2:**
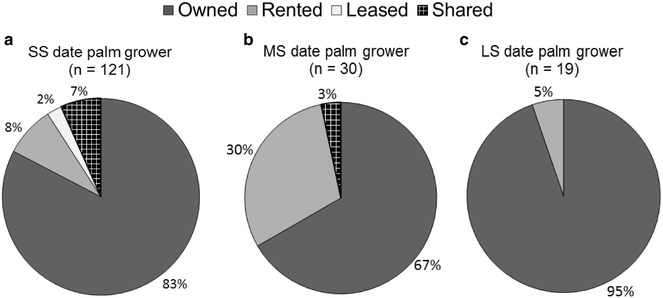
**a**, **b**, **c** Land ownership characterization of small scale (SS), medium scale (MS) and large scale (LS) date palm growers in four provinces (Punjab, Khyber Pakhtunkhwa, Sindh, and Baluchistan) of Pakistan interviewed during 2012–2013

On average, SS date palm growers owned and leased more arable land (212 ± 36 ha), they were also sharing land for the cultivation of other crops and fruit trees, followed by MS (22 ± 15 ha) and LS date palm growers (18 ± 7 ha). More MS growers rented land than SS and LS growers did (Fig. 2a–c).

### Household income

Agriculture was the main source of income for date palm growers, occasionally coupled with off-farm income (job, business, and labour). Among surveyed HHs, 24 % were totally dependent on dates and by-products for their living. Most of the HH earned <500 US$ per month and only 18 % had a monthly income from 1000 to ≤2000 US$ whereby date sales contributed >50 % to total income of 39 % HHs (Table 4). Less than 20 % HHs had a monthly income of ≥2000 US$, among them >10 % were earning it from dates (Table 4).

**Table 4:** Income characteristics of 170 date palm growers in four provinces (Punjab, Khyber Pakhtunkhwa, Sindh and Baluchistan) of Pakistan, interviewed during 2012–2013

Variable	Frequency (n)	Percentage
*Occupation*		
Agriculture	95	56
Agriculture + government job	15	9
Agriculture + labour	17	10
Agriculture + business	29	17
Agriculture + private job	14	8
*Estimated monthly income (US$)*		
≤100	4	2
101–200	14	8
201–300	29	17
301–500	43	25
501–1000	20	12
1001–2000	30	18
>2000	30	18
*Estimated monthly expenses (US$)*		
≤100	9	5
101–200	33	19
201–300	26	15
301–500	30	18
501–1000	14	8
1001–2000	53	32
>2000	5	3
*Date palm share in income (%)*		
≤10	56	33
11–30	23	14
31–50	24	14
51–70	12	7
71–90	14	8
91–100	41	24
*Estimated monthly date palm sale (US$)*		
≤100	77	45
101–200	26	15
201–300	3	2
301–500	10	6
501–1000	4	2
1001–2000	32	19
>2000	18	11

Different cultivation practices were observed in the surveyed areas. Most of the HHs (85 %) had palm groves. This trend was more pronounced in MS growers and was rare in LS and SS producers whose palms grew scattered. 14 % of the surveyed HHs intercropped date palm with different crops such as cereals, legumes, and fruit trees. This practice was common in LS holdings while only SS growers grew dates in home gardens (Table 5).

**Table 5:** Farm type and usage of date palm fruit and by-products of small scale (SS), medium scale (MS) and large scale (LS) palm growers in four provinces (Punjab, Khyber Pakhtunkhwa, Sindh, and Baluchistan) of Pakistan, interviewed during 2012–2013

Variable	SS grower (n = 121)	MS grower (n = 30)	LS grower (n = 19)
*Farming type*			
Groves	57	83	47
Scattered trees	34	3	0
Intercropped	5	7	32
Home garden	4	0	0
*Household usage*			
Fruit as food	100	97	100
Fruit as animal feed	50	43	42
Fuel from frond	39	27	26
Mats from frond	32	43	58
Huts from stem and frond	13	27	37
Hand fans from frond	9	13	5
Staple dishes from frond	11	7	16
Suckers	100	97	100
*Commercial usage*			
Fruit as food	61	100	100
Fruit as animal feed	2	0	0
Mats from frond	25	30	21
Huts from stem and frond	1	13	0
Hand fans from frond	3	7	0
Staple dishes from frond	14	3	5
Suckers	29	50	37

Date palm usage differed among growers. Almost all HHs used dates as food for their families and also sold dates. Most farmers used low quality dates as animal feed at the household and commercial level. Usage of palm by-products for household and commercial purposes varied from farmer to farmer (Table 5). Farmers regularly separated suckers from good palm varieties and either grew them separately in their own grove or sold them at an average price of 8 US$ per sucker.

### Date value chains

**Fig. 3:**
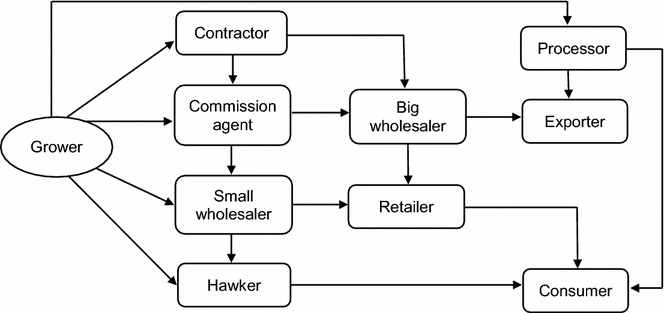
Structure of date palm value chain in four provinces (Punjab, Khyber Pakhtunkhwa, Sindh, and Baluchistan) of Pakistan during 2012–2013

Generally, palm growers preferred to sell fruits fresh because of scarce availability of labour, storage houses, and processing facilities. However, especially LS growers tended to process their dates prior to selling; they either made *chuhara*—a special type of dried date which is prepared by boiling premature fruits and adding rang kat (sodium formaldehyde)—or they sold dried dates. In Punjab, Sindh and Baluchistan most palm growers sold their dates to contractors, commission agents or wholesalers (Fig. 3). Many HHs were bound to sale their dates to commission agents, because of the already taken loans from them with a 10–11 % interest rate compared with agricultural banks charging 13–15 %. It was observed that contractors often knew more about date sale, resource use, and market access than growers. Date prices on wholesale markets were controlled by wholesalers rather than by the government. In the date market chain, wholesalers work as intermediaries between growers/contractors and retailers/buyers. Exporters were either commission agents or owners of processing units and were mainly selling *chuhara* to India. Farmers gave various reasons for entering a contract in which they agreed to sell the entire production of their grove to an intermediary: particularly prominent was distance to the next market (Table 6). The results also illustrate that only SS and MS palm growers traded with commission agents while MS sold fruits to hawkers, markets, and directly to consumers. SS date palm growers used a big portion of their dates for self-consumption (Table 6). Hawkers were buying dates directly at the farm gate or from small wholesalers within the district and moved from street to street to sell them. Most retailers who owned small nut shops bought dates from small wholesalers within the district while retailers who owned hypermarkets or superstores purchased dates from big wholesalers within provinces (Fig. 3).

**Table 6:** Marketing of dates of 170 small scale (SS), medium scale (MS) and large scale (LS) palm growers in four provinces (Punjab, Khyber Pakhtunkhwa, Sindh, and Baluchistan) of Pakistan, interviewed during 2012–2013

Variable	SS grower (n = 121)	MS grower (n = 30)	LS grower (n = 19)
*Consumer/buyer of dates*			
Contractor	12	37	32
Wholesaler	14	43	68
Commission agent	32	11	0
Retailer	0	0	0
Hawker	0	3	0
Directly to consumer	0	3	0
Market	0	3	0
Self-consumption	42	0	0
*Reasons for contract*			
Shortage of money	26	33	42
Distance to market	54	27	37
Shortage of labour	10	13	5
Lack of time	10	27	16

Based on the two-step cluster analysis date palm growers of four provinces (Punjab, Sindh, KPK and Baluchistan) were divided into four groups to better compare socio-economic differences among date palm growers of such areas. HHs invested an average of 2597 US$ on the transportation of dates and date-by-products to the market (Table 7). Thereby the majority of date palm growers from Sindh did not own store houses nor did they have vehicles to transport their produce to the market. Average number of bunches of dates per palm was highest in palm cultivars grown in Baluchistan while average weight of fruit per bunch was significantly higher in the fields of KPK growers (Table 7). Farmers received the highest average sales price of 1.22 US$ per kg for fresh dates and 1.28 US$ per kg for *chuhara* because of the high demand for such fruits. In contrast, average sales price of dried dates was only 0.56 US$ per kg.

**Table 7:** Key characteristics of major variables distinguished by 170 date palm growers in four provinces (Punjab, Khyber Pakhtunkhwa, Sindh, and Baluchistan) of Pakistan interviewed during 2012–2013

Variable	Punjab date palm grower (n = 60)	KPK date palm grower (n = 20)	Baluchistan date palm grower (n = 50)	Sindh date palm grower (n = 40)	P*
Transport expenditure (US$)	516 ± 430.14	100 ± 0.510	139 ± 107.264	2769 ± 2001	≤0.096
Storage expenditure (US$)	48 ± 0	15 ± 0	500 ± 92.231	601 ± 132	≤0.088
Annual sale (US$)	317 ± 1158	10304 ± 8036	2123 ± 1523	24115 ± 6312	≤0.001
Mature date palms	74 ± 194	385 ± 360	175 ± 64	1216 ± 562	≤0.001
Yield (number of bunches date palm^−1^)	12 ± 1.671	13 ± 1.261	17 ± 2.255	16 ± 2.554	≤0.124
Weight of bunch (kg)	6 ± 1.098	15 ± 1.341	11.48 ± 1.265	14 ± 1.918	≤0.093
Price of fresh date kg^−1^ (US$)	0.701 ± 0.51	2.48 ± 0.51	0.841 ± 0.301	0.871 ± 0.23	≤0.402
Price of dried date kg^−1^ (US$)	0.961 ± 0	0 ± 0	0.691 ± 0.261	0.591 ± 0.101	≤0.322
Price of *chuhara* kg^−1^ (US$)	0.916 ± 1.311	2.181 ± 0.591	1.531 ± 0.061	0.531 ± 0.181	≤0.191

Data show means ± one standard deviation

P = probability/significance value

* Kruskal–Wallis test

### Cropping practices

**Fig. 4:**
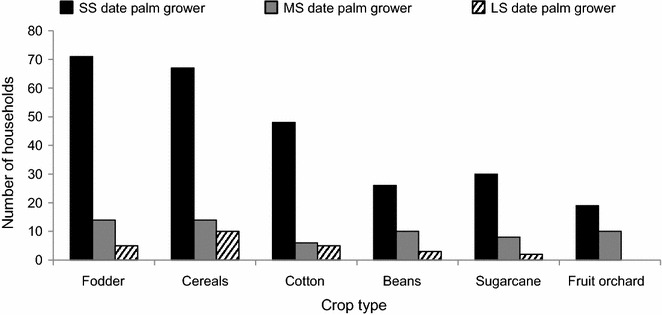
Major crops grown by different categories of small scale (SS), medium scale (MS) and large scale (LS) date palm growers in four provinces (Punjab, Khyber Pakhtunkhwa, Sindh, and Baluchistan) of Pakistan interviewed during 2012–2013

Date palm growers co-cultivated a diversity of crops and fruits (Fig. 4). More than 50 % of date palm growers regularly sowed rice and wheat in their fields. Most SS palm growers cultivated fodder crops such as berseem (*Trifolium alexandrinum* L., 40 %), alfalfa (*Medicago sativa* L., 35 %), sorghum (*Sorghum bicolor* Moench., 36 %), maize (*Zea mays* L., 70 %), and mustard (*Brassica* spp., 50 %), either to be used by their own livestock or for sale to other farmers and dairy farms. SS and MS farmers also grew other fruits such as mango (*Mangifera indica* L., 8 %), ber (*Ziziphus mauritiana* Lam., 3 %), citrus (4 %), guava (*Psidium guajava* L., 5 %), and pomegranate (*Punica granatum* L., 2 %). While in Punjab mangoes predominated, in Sindh there was more banana (*Musa* spp.).

**Fig. 5:**
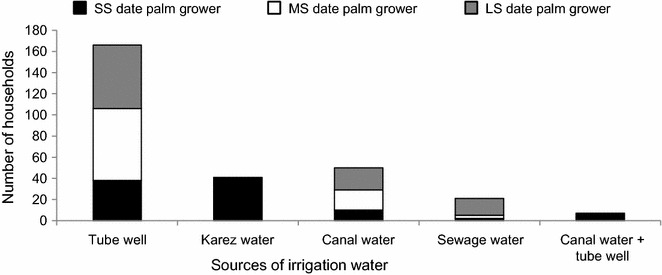
Major sources of irrigation water used by different categories of small scale (SS), medium scale (MS) and large scale (LS) date palm growers in four provinces (Punjab, Khyber Pakhtunkhwa, Sindh, and Baluchistan) of Pakistan interviewed during 2012–2013

Depending on holding size, palm growers irrigated their crops with well water, canal water or a combination of both. In water scarce areas irrigation depended on sewage water and the majority of SS palm growers from Baluchistan used traditional Karez irrigation systems, in which gravity-dependent underground channels carry water from mountain springs to agricultural fields (Fig. 5).

More SS than LS or MS date growers drilled their crops, however, ridge sowing was also very common in the study area. The results also indicate that most HHs used chemical fertilizers (85 %), while farm yard manure (FYM) was only used by date growers who also kept livestock (55 %). Most of the SS palm growers applied pesticides on their crops while this practice was less prevalent in MS and LS growers (Table 8).

**Table 8:** Use of pesticides, fertilizers, and farmyard manure (FYM) of small scale (SS), medium scale (MS) and large scale (LS) date palm growers in four provinces (Punjab, Khyber Pakhtunkhwa, Sindh, and Baluchistan) of Pakistan interviewed during 2012–2013

Variable	SS growers (n = 121)	MS growers (n = 30)	LS growers (n = 19)	χ^2^	P
*Use of pesticide*					
Yes	69	47	42	9.038	≤0.011
No	31	53	58		
*Use of fertilizer*					
Yes	98	77	79	18.484	≤0.001
No	2	23	21		
*Use of FYM*					
Yes	66	63	37	6.010	≤0.050
No	34	37	63		

### Grower constraints

**Fig. 6:**
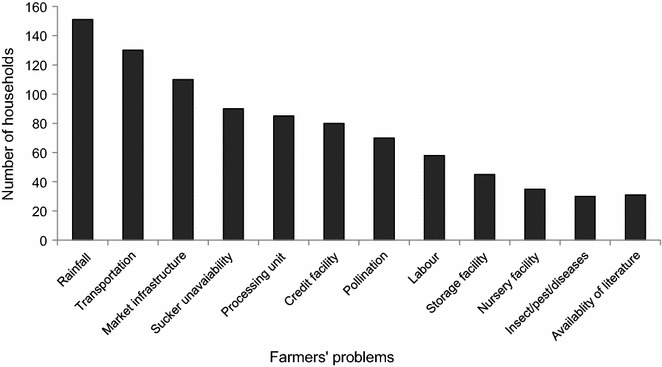
Major problems experienced by 170 date palm growers in four provinces (Punjab, Khyber Pakhtunkhwa, Sindh, and Baluchistan) of Pakistan interviewed during 2012–2013

Palm cultivation and management in the surveyed areas faced a range of constraints (Fig. 6). Most farmers reported problems with monsoon rainfall during the fruit ripening season (June–October) which usually destroyed fruits within 2–3 days by causing cracks in the epicarp. More than 50 % of HHs complained about deficits in market infrastructure, while unavailability of credit, labour scarcity, transportation deficiency and lacking nurseries, processing plants, and storage houses were other important problems. Family members of most SS growers were working in family fields performing different tasks for which they were hardly paid. Most growers sun-dried their dates due to unavailability of alternative drying and processing units. A large number of the respondents reproduced date palms from suckers of their own cultivars and lacked access to elite germplasm for quality production. Growers had local names for most of their date cultivars and were often unaware of their commercial name (Table 9). Manual pollination was difficult for >60 % farmers because of unavailability of male pollen and of skilled labour.

**Table 9:** Different date palm cultivars grown by 170 date palm growers in four provinces (Punjab, Khyber Pakhtunkhwa, Sindh, and Baluchistan) of Pakistan interviewed during 2012–2013

HHs	Cultivars	% of cultivars
N^a^ = 1	Ajwa, Akhrot, Amber, Angoor, Barni, Begum Jhangi, Berehmi, Doki, Kalma, Kobra, Meeri, Patal, Pathri, Shershai, Zeerin	38.5
N^a^ = 2–6	Basra, Chohara, Daanda, Dandari, Halawi, Ketchen, Khudrawi, Kupra, Pori, Rati, Sabzo, Sawi, Shamran, Sufaida, Sundri, Zaidi	41.0
N^a^ > 6	Aseel, Desi, Dhakki, Haleni, Karbalaen, Karoch, Koharba, Muzawati	20.5

^a^Number of date palm cultivars grown by the households

## Discussion

### Grower characteristics

The 8 ha average farm size of our date producers was similar to the 6 ha reported by Jalal-Ud-Din (2011) for cropping systems in the Mardan district, Pakistan, but average HH size in our study exceeded the 4 members reported by Shrinivasan (2012) for rural India which reflects the more extended family structure in Pakistan (Hagmann 2012). In the current study, the literacy level of palm growing HH heads was low compared with the results of Javed et al. (2008) who concluded that 82 % of the respondents in Faisalabad had 5 years and 18 % had 10 years of formal education which may facilitate adoption of modern farming practices (Burchi and Muro 2007).

### Household income

The majority of the date growers in our study area had <150 mature palms and were thus small scale farmers. Many authors claim that >70 % of the world’s food especially in developing countries is produced by small scale farmers which underlines their role for food security maintenance and in preserving complex crop–livestock systems (Berry and Cline 1979; Carter 1984; Cornia 1985; Heltberg 1998).

Average annual income of date producers in our study area was 6000 US$ which is much larger than the average annual income of 1475 US$ reported for palm growers in the Southern Punjab (Ata et al. 2014). For almost half of the farmers of the present study, date sales contributed 50 % to total HH income. Chowdhury et al. (2008) also reported a significant contribution of date palm to the total income of farmers in date producing areas of rural Bangladesh. The low contribution of off-farm activities to date palm grower’s HH income is similar to results of Ata (2011) for the Pakistani Southern Punjab where crop sale was the primary and livestock rearing the secondary income source. All respondents in our study area used dates as a food crop and most utilised other palm parts (especially fronds and trunks) to make mates, staple dishes, and huts for both household and commercial purposes. Hassan et al. (2006) reported the same usage of date palm in the Punjab where dates and by-products were used and sold by the native community. Most of the respondents in the Jhang District (Punjab) also used dates as animal feed. Such type of usage is also reported by Walsborn (2008) in Iraq where low quality dates are sold as animal feed to sheep herders, dairies, and to the companies making date syrup. This practice should also be adopted in other date palm growing areas of Pakistan to increase HH income.

### Date value chain

The main reason for farmers taking informal loans from commission agents was lack of appropriate government-based credit schemes, a situation similar to that reported by Ata (2011). Local banks demanded land as a guarantee for loans which many farmers felt overburdened by. A similar situation was reported by Mcegypt (2014) who analysed date value chains in Aswan (Egypt), where agricultural development banks were charging a 15–17 % interest rate from farmers. In our study area, some commission agents had shops in big wholesale markets where they allowed growers to sell their dates in their shops on the condition that they will get 7 % of the total sale (Ata 2011). This was similar to palm growers in Gaza (Palestine) who paid 6 % of the value of their date sales to wholesalers to sell their produce (RUAF 2014).

Contractors are important parts of the date marketing chain in Pakistan. They perform early yield estimates of palm groves and costs for labour, storage, transportation, and processing of dates (Khushk et al. 2009). Often contracts for date palm grove were made as early as at the pollination stage. This again was similar to data reported by RUAF (2014) for value chain of dates in Gaza. In our study contractors payed (5 %) more to date palm growers for their produce compared with commission agents. Some contractors also took loans from commission agents; they either sold dates through them or payed back loans and interest after marketing their produce. Aujla et al. (2007) noted that 70–90 % of the fruit growers in Pakistan sold their harvesting rights at the flowering stage to contractors, while more than 97 % of the fruit contractors took loans from commission agents to pay the contract money to fruit orchard owners and labourers working in the groves. This underlines the dependency of contractors on commission agents for credit in the study area.

Often farmers travelled far to self-market their produce at higher rates compared with those obtained at the farm gate, but when they reached the market place wholesale prices of dates had fell. In the absence of government-fixed prices, wholesalers controlled prices according to offer and demand. Aujla and Jagirani (2002) reported that the main problem in marketing of horticultural crops in Pakistan are sudden price fluctuations indicating oligopolistic effects on the demand side (Hine and Ellis 2001).

In our study area, processing and export were not part of a regular marketing chain. A similar situation was reported by Walsborn (2008) for date marketing in Iraq. In our study, 69 % of the date palm growers sold their produce to contractors, wholesalers or commission agents, mainly due to the unavailability of proper credit facilities and labour, and the large distance to the nearest market. The low revenues for the producer is a general characteristic of the fruit marketing sector in Pakistan, where farmers’ share of the consumer price is about one-fourth while the remaining share goes to commission agents (7 %), contractors (39 %), wholesalers (9 %) and retailers (19 %; Mahmood et al. 1989; Khushk and Smith 1996).

Our study indicates only little value addition through grading, sorting, and processing. At the date grower’s level there were large value losses due to bird attacks, molds following monsoon rains, improper packing, and poor transport to the market. Overall, improper post-harvest handling led to major losses in produce quality. Walsborn (2008) reported the same for date palm groves of Iraq where farmers were not sorting and grading dates after harvest. In the current study, most growers threw harvested date bunches to the ground. This not only destroyed dates but also contaminated fruits with dust and sand, which was affecting their quality. Walsborn (2008) observed similar problems at date harvesting in Iraq and Elshafia and Salih (1984) showed that harvesting by ropes reduced fruit losses up to 45 %.

### Cropping practices

Most date growers also engaged in cereal, fodder and cotton production, and the majority of HHs retained their cereals for home consumption. Similar results were reported by Singh et al. (2011) for the Indian Punjab, where rice–wheat cropping predominates. Contrasting results were shown by Jacobi et al. (2009) in India, where the majority of the HHs preferred short duration crops such as leafy vegetables to obtain higher profits per unit area compared with grain crops, however, their study took place in peri-urban areas. More than 80 % of the HHs in our study area had palm groves in their fields, which is different from the findings of Ata (2011) from Southern Punjab, who reported that only 2.5 % of respondents had groves and 97.5 % had scattered date palm populations. In our study, less than 15 % of the HHs intercropped date palms with other crops and fruits. Henrik et al. (2004) supported by Abouziena et al. (2010) stated that intercropping has potential for generating more stable yields, due to self-regulation effects in crops and balancing of farm income. To what degree this is also the case in Pakistani date palm systems merits further study. It is nevertheless clear that date palm production systems in Pakistan still have great potential for improvement.

Our study shows that date palm growers in Bahawalpur used sewage water for irrigation. Though this enhances the nutrient supply to date palm groves there may be health concerns, particularly at harvest when dates get in direct contact with the irrigation water loaded with microbes. In our study, 85 % of the farmers used mineral fertilizers, while the use of FYM was low (55 %). Enhanced use of FYM may be an effective way to decrease production costs but is in conflict with widespread manure use as poor people’s fuel.

### Grower constraints

In our study, average fruit weight per date palm was 90 kg of which reportedly more than half was destroyed by rain, wind and birds. Hence harvested yields were substantially smaller. Ata (2011) and Mcegypt (2014) also reported date yields of 50–90 kg per palm, depending on the date variety and environmental conditions in Punjab and Aswan (Egypt). Yields of Pakistani and Egyptian date palm groves are lower than that those reported by RUAF (2014) for palm cultivars grown in Gaza which were producing an average yield of 150 kg dates per tree. Mauk and Sharabeen (2005) reported that Deglet Noor cultivars grown in California (USA) have the potential to produce 123 kg of dates per tree at intensive fertilization and manual pollination. A report by USAID (2008) from the Inma agribusiness program in Iraq claims that manual pollination of date palm has the potential to increase fruit yield by 60–80 kg per tree.

Only a few farmers in Punjab used improved palm varieties in intensively managed groves, which indicate the widespread marginal nature of date production. Interesting was that the locally used date cultivars were named either on the basis of the fruit shape (“Angoor” = grapes and “Akhrot” = walnut), on the basis of their color (“Sawi”/“Sabzo” = green date and “Rati” = red date) or on the basis of their origin (“Basra” and “Karbalaen”) rather than fruit taste, preferred use or organoleptic properties. Mirbahar et al. (2014) reported that date palm cultivars currently used in Pakistan are the result of long-lasting, continuous selection by the farmers which were given folk names, but these were not consistent across locations (Kumar 2011). Similar to the study of Ata (2011) in Dera Ghazi Khan and Muralidharan and Baidiyavadara (2013) in India germplasm exchange was rare among palm growers.

## Conclusions

The widespread use of diverse date palm products in the study area indicates the role of this crop for local farm HHs. The low literacy rate of farmers may hinder the adoption of farm innovations leading to more intensive production systems and post-harvest value addition for this crop. Improved market access, cooperative sale structures and/or quality-based labeling with direct sales to consumers may help to enhance the role of dates in HH income thereby strengthening local farmers’ income. This will require enhanced government investments in rural extension, consumer and producer awareness of produce quality including the establishment of grading standards for dates and palm nurseries with high quality germplasm. Generalizing the results of our study is hampered by the fact that the selection of our HHs based on the snowball sampling approach was not fully random given lacking governmental or private records of date palm growers in most remote study areas.

## References

[CR1] Abas Z, Ragkos A, Mitsopoulosn I, Theodoridis A (2013). The environmental profile of dairy farms in Central Macedonia (Greece). Proc Technol.

[CR2] Abdulkadir A, Dossa LH, Lompo DJP, Abdu N, Keulen HV (2012). Characterization of urban and peri-urban agroecosystems in three West African cities. Int J Agric Sustain.

[CR3] Abouziena HFH, Elham ZAE, Youssef RA, Sahab AF (2010). Efficacy of intercropping mango, mandarin or Egyptian clover plants with date palm on soil properties, rhizosphere microflora and quality and quantity of date fruits. J Am Sci.

[CR4] Al-Shahib W, Marshall RJ (2003). The fruit of date palm: its possible use as best food for the future?. Int J Food Sci Nutr.

[CR5] Anwar MA (2006). *Phoenix dactylifera* L: a bibliometric study of the literature on date palm. Malays J Libr Inf Sci.

[CR6] Ata S (2011) A study of date palm market chain and its role in food security and livelihoods of farmers in the South Punjab. M.Sc. (Hons.) thesis, University of Agriculture, Faisalabad

[CR7] Ata S, Shahbaz B, Ahmad M, Khan IA (2012). Factors hampering date palm production in the Punjab: a case study of D. G. Khan district. Pak J Agric Sci.

[CR8] Ata S, Shahbaz B, Khan IA, Iftikhar M (2014). Role of date palm in livelihoods of farmers of marginal areas: a case study of South Punjab, Pakistan. J Agric Res.

[CR9] Aujla KM, Jagirani AW (2002) Production and marketing of potatoes in Pakistan: opportunities and constraints. Socioeconomics Research Studies 2001–02, Social Sciences Division. Pakistan Agricultural Research Council, Islamabad

[CR10] Aujla KM, Abbas M, Mahmood K, Saadullah S (2007). Marketing system of fruits, margins and export potential in Pakistan. Pak J Life Soc Sci.

[CR11] Beinroth FH, Khan A, Nizami MI, Syal MN (1985) Soil taxonomy and agrotechnology transfer. In: Ahmad M, Akram M, Baig MS, Javed MY, Amin R (eds) Proceedings of the XII international forum on soil taxonomy and agrotechnology transfer. Lahore, Pakistan. Soil Management Support Services, USA, pp 199–230

[CR12] Berry RA, Cline WR (1979). Agrarian structure and productivity in developing countries.

[CR13] Bhansali RR, Ramawat KG (2010). Date palm cultivation in the changing scenario of Indian arid zones. Desert plants: biology and biotechnology.

[CR14] Burchi F, Muro PD (2007) Education for rural people, a neglected key to food security. A cross country analysis. Working paper. Department of Economics, Roma Tre, Università degli Studi, pp 1–45

[CR15] Carter MR (1984). Identification of the inverse relationship between farm size and productivity: an empirical analysis of peasant agricultural production. Oxf Econ Pap.

[CR16] Chao CCT, Krueger RR (2007). The date palm (*Phoenix dactylifera* L.): overview of biology, uses and cultivation. Hortic Sci.

[CR17] Chowdhury MSH, Halim MA, Muhammed N, Haque F, Koike M (2008). Traditional utilization of wild date palm (*Phoenix sylvestris*) in rural Bangladesh: an approach to sustainable biodiversity management. J For Res.

[CR18] Cornia GA (1985). Farm size, land yields and the agricultural production function: an analysis for fifteen developing countries. World Dev.

[CR19] Dossa LH, Abdulkadir A, Amadou H, Sangare S, Schlecht E (2011). Exploring the diversity of urban and peri-urban agricultural systems in Sudano-Sahelian West Africa: an attempt towards a regional typology. Landsc Urban Plan.

[CR20] Dossa LH, Sangaré M, Buerkert A, Schlecht E (2015). Intra-urban and peri-urban differences in cattle farming systems of Burkina Faso. Land Use Policy.

[CR21] El Hadrami A, Al Khayri JM (2012). Socioeconomic and traditional importance of date palm. Emir J Food Agric.

[CR22] Elshafia A, Salih SM (1984) Estimation of harvest, transport and storage losses in some Sudanese fruits and vegetables. FRC. Annual report

[CR23] FAO, Food and Agriculture Organization of the United Nations (2014) Food and agricultural commodities production for Pakistan for 2012. www.faostat.fao.org/DesktopDefault.aspx?PageID=339&lang=en&country=165. Accessed 24 July 2014

[CR24] Goletti F, Samman E (1999) The importance of the post-production sector to sustainable rural livelihoods. In: The Importance of post-production to sustainable rural livelihoods, GASGA proceedings, vol, 11. Natural Resources Institute University of Greenwich, Chatham, pp 9–20

[CR25] Goodman LA (1961). Snowball sampling. Ann Math Stat.

[CR26] Hagmann J (2012) Opportunities and constraints of peri-urban buffalo and dairy cattle systems in Faisalabad, Pakistan. ICCD working paper (21). University of Kassel, Kassel

[CR27] Hassan S, Bakhsh K, Gill ZA, Maqbool A, Ahmad W (2006). Economics of growing date palm in Punjab, Pakistan. Int J Agric Biol.

[CR28] Heltberg R (1998). Rural market imperfections and the farm size-productivity relationship: evidence from Pakistan. World Dev.

[CR29] Henrik NH, Kinane J, Knudsen MT, Jensen ES (2004) Intercropping and sustainability. www.orgprints.org/3132/. Accessed 7 Sept 2015

[CR30] Hine JL, Ellis SD (2001). Agricultural marketing and access to transport services.

[CR31] Jacobi J, Drescher AW, Amerasinghe PH, Weckenbrock P (2009). Agricultural biodiversity: strengthening livelihoods in peri-urban Hyderabad, India. Urban Agric Mag.

[CR32] Jain SM (2012). Date palm biotechnology: current status and prospective—an overview. Emir J Food Agric.

[CR33] Jalal-Ud-Din M (2011). The socio-economic problems of small farmers in adopting new agricultural technologies: a case study of three villages in district Mardan. Sarhad J Agric.

[CR34] Javed ZH, Khilgi BA, Mujahid M (2008). Impact of education on socio-economic status of villager’s life. Pak Econ Soc Rev.

[CR35] Khiari R, Mauret E, Belgacem MN, Mhemmi F (2011). Tunisian date palm rachis used as an alternative source of fibers for papermaking applications. BioResources.

[CR36] Khushk AM, Smith LED (1996). A preliminary analysis of the marketing of mango in Sindh Province, Pakistan. Pak Dev Rev.

[CR37] Khushk AM, Memon A, Aujla KM (2009). Marketing channels and margins of dates in Sindh, Pakistan. Pak J Agric Res.

[CR38] Kumar S (2011) Advantages and disadvantages of common names and botanical name. www.ehomoeopathy.wordpress.com/2011/10/21/advantages-and-disadvantages-of-common-names-botanical-name/. Accessed 7 Sept 2015

[CR39] Mahmood K, Khan SM, Afzal M (1989) Production and marketing of potatoes in upland Balochistan: a preliminary survey. MART/AZR research report 45. ICARDA, Pakistan

[CR40] Mahmoudi H, Hosseininia G, Azadi H, Fatemi M (2008). Enhancing date palm processing, marketing and pest control through organic culture. J Org Syst.

[CR41] Malik MN (1994). Horticulture.

[CR42] Mauk P, Sharabeen I (2005) Sample costs to establish a date palm orchard and produce dates in the Coachella Valley, Riverside County, 2005–2006. University of California Cooperative Extension (UCCE). www.coststudyfiles.ucdavis.edu/uploads/cs_public/b5/55/b5553ac8-9aaa-49e1-b617-8e87f0e55bf0/dates_si_2005.pdf. Accessed 13 Aug 2015

[CR43] Mcegypt (2014) Dates in Aswan, value chain analysis. www.mcegypt.me/wp-content/uploads/2014/01/Dates-Value-Chain-in-Aswan.pdf. Accessed 20 July 2015

[CR44] Mirbahar AA, Markhand GS, Khan S, Abul-Soad AA (2014). Molecular characterization of some Pakistani date palm (*Phoenix dactylifera* L.) cultivars by RAPD markers. Pak J Bot.

[CR45] Muralidharan CM, Baidiyavadara DA (2013) Variability and diversity of elite date palm *Phoenix dactylifera* L. in date groves of Kachchh (Gujarat) India. In: Proceeding of 1st international symposium on date palm. Acta Horticulturae 994:263–270

[CR46] Nixon RW (1934). Metaxenia in dates. Proc Am Soc Hortic Sci.

[CR47] Nixon RW (1936). Metaxenia and interspecific pollinations in *Phoenix*. Proc Am Soc Hortic Sci.

[CR48] Nixon RW, Carpenter JB (1978) Growing dates in the United States. Bulletin no. 207. United States Department of Agriculture, Washington, DC

[CR49] PHDEB, Pakistan Horticulture Development and Export Board (2008). Dates marketing strategy. www.phdec.org.pk/MktStrategies/Dates.pdf. Accessed 5 Aug 2014

[CR50] PMD, Pakistan Meteorological Department. www.namc.pmd.gov.pk/agromet-bulletins.php#. Accessed 12 Feb 2015

[CR51] Raza MA, Younas Mm Buerkert A, Schlecht E (2014). Ethno-botanical remedies used by pastoralists for the treatment of livestock diseases in Cholistan desert, Pakistan. J Ethnopharmacol.

[CR52] Rehman SU, Predotova M, Khan IA, Schlecht E, Buerkert A (2013). Socio-economic characterization of integrated cropping systems in urban and peri-urban agriculture of Faisalabad, Pakistan. J Agric Rural Dev Trop Subtrop.

[CR53] RUAF, Resource Centre for Urban Agriculture and Forestry (2014) Palm dates value chain report. www.ruaf.org/sites/default/files/PALM%20DATE%20VC%20%20FINAL.pdf. Accessed 21 July 2015

[CR54] Shrinivasan R (2012) Median household size drops below 4 in cities. The Times of India. www.articles.timesofindia.indiatimes.com/India/31236370_1_household-family-size-census. Accessed 20 July 2015

[CR55] Singh S, Park J, Litten-Brown J (2011) The economic sustainability of cropping systems in Indian Punjab: a farmer’s perspective. Paper prepared for presentation at the EAAE 2011 Congress Change and Uncertainty Challenges for Agriculture, Food and Natural Resources. ETH, Zurich

[CR57] Suleri AQ, Haq S (2009) Food insecurity in Pakistan. www.documents.wfp.org/stellent/groups/public/documents/ena/wfp225636.pdf. Accessed 23 July 2015

[CR58] Swingle WT (1928). Metaxenia in the date palm, possibly a hormone action by the embryo or endosperm.

[CR59] USAID, United States Agency for International Development (2008) Inma agribusiness program on dates value chain analysis and opportunities for Iraq. www.inma-iraq.com/sites/default/files/tr_dates_value_chain_july08.pdf. Accessed 14 July 2015

[CR60] Vazhacharickal PJ, Predotova M, Chandrasekharam D, Bhowmik S, Buerkert A (2013). Urban and peri-urban agricultural production along railway tracks: a case study from the Mumbai Metropolitan Region. J Agric Rural Dev Trop Subtrop.

[CR61] Walsborn R (2008) Date sector report and value chain development program. www.pdf.usaid.gov/pdf_docs/PNADP536.pdf. Accessed 22 July 2015

[CR62] Zaid A, de Wet PF (2002) Date palm cultivation, Pollination and bunch management. Plant Production and Protection, Paper no. 156. FAO, Rome, pp 145–175

[CR63] Zohary D, Hopf M (2000). Domestication of plants in the old world: the origin and spread of cultivated plants in West Asia, Europe and the Nile Valley.

